# Plastic deformation of directionally solidified ingots of binary and some ternary MoSi_2_/Mo_5_Si_3_ eutectic composites

**DOI:** 10.1080/14686996.2016.1218248

**Published:** 2016-09-08

**Authors:** Hirotaka Matsunoshita, Yuta Sasai, Kosuke Fujiwara, Kyosuke Kishida, Haruyuki Inui

**Affiliations:** ^a^ Department of Materials Science and Engineering, Kyoto University, Kyoto, Japan; ^b^ Center for Elements Strategy Initiative for Structural Materials (ESISM), Kyoto University, Kyoto, Japan

**Keywords:** Transition-metal silicides, microstructure, mechanical properties, high-temperature deformation, 10 Engineering and Structural materials, 103 Composites, 302 Crystallization / Heat treatment / Crystal growth, 503 TEM, STEM, SEM

## Abstract

The high-temperature mechanical properties of directionally solidified (DS) ingots of binary and some ternary MoSi_2_/Mo_5_Si_3_ eutectic composites with a script lamellar structure have been investigated as a function of loading axis orientation and growth rate in a temperature range from 900 to 1500°C. These DS ingots are plastically deformed above 1000 and 1100 °C when the compression axis orientations are parallel to [1$\bar{1}$0]_MoSi2_ (nearly parallel to the growth direction) and [001]_MoSi2_, respectively. [1$\bar{1}$0]_MoSi2_-oriented DS eutectic composites are strengthened so much by forming a script lamellar microstructure and they exhibit yield stress values several times higher than those of MoSi_2_ single crystals of the corresponding orientation. The yield stress values increase with the decrease in the average thickness of MoSi_2_ phase in the script lamellar structure, indicating that microstructure refinement is effective in obtaining better high-temperature strength of these DS eutectic composites. Among the four ternary alloying elements tested (V, Nb, Ta and W), Ta is found to be the most effective in obtaining higher yield strength at 1400 °C.

## Graphical abstract

**Figure:**
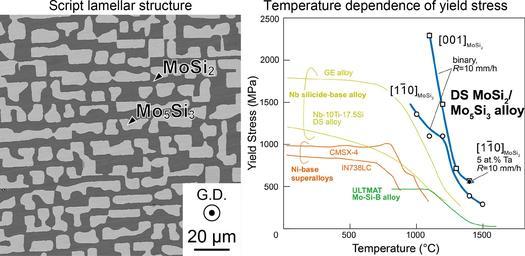

## 1. Introduction

There is an increasing demand for ultrahigh-temperature structural materials that can drastically raise the operating temperature of combustion systems in fossil-fueled power plants so as to improve their thermal efficiency and consequently to reduce fuel consumption as well as climate-warming CO_2_ emission. Since the turbine inlet temperature of the most advanced gas turbine combustion systems already exceeds 1600 °C, which is about 200 °C higher than the melting temperature of current Ni-based superalloys,[1] Ni-based superalloy turbine blades are usually used with air cooling. Therefore, a drastic increase in thermal efficiency of these gas turbine combustion systems is unlikely, unless a new class of ultrahigh-temperature structural materials is developed, which can withstand severe environments and exhibit superior mechanical properties and corrosion resistance at high temperatures. MoSi_2_-based alloys and composites have received a considerable amount of attention as promising candidates for such ultrahigh-temperature structural applications due to their high melting temperatures (around 2000 °C), low densities, high thermal conductivities and good oxidation resistance.[2–12] Various MoSi_2_-based composites produced by powder metallurgy routes have been investigated extensively.[2–6,8,9,12] Some of these MoSi_2_-based composites exhibit improved fracture toughness at ambient temperature. However, their high-temperature strength is rather poor mostly because of intergranular SiO_2_ layers.[2,4,13] Alternatively, directionally solidified (DS) MoSi_2_-based eutectic composites containing the Mo_5_Si_3_ phase with the tetragonal D8_m_ structure have been confirmed to exhibit better creep properties than other MoSi_2_-based composites produced through powder-metallurgy routes due mainly to the elimination of high-angle grain boundaries and intergranular SiO_2_ layers.[14] DS MoSi_2_/Mo_5_Si_3_ eutectic composites have a fine script lamellar microstructure composed of a continuous MoSi_2_ matrix and an interconnected network of Mo_5_Si_3_ elongated along the growth direction.[15–17] Mechanical properties of DS eutectic composites are expected to be affected by microstructural characteristics such as lamellar spacing, orientation relationship and interface morphology as well as alloying elements. Further improvements in high-temperature strengths are thus expected to be highly probable by controlling the growth conditions in the DS process and also by ternary additions. Recently, we have investigated effects of ternary additions on the microstructure and thermal stability of DS MoSi_2_/Mo_5_Si_3_ eutectic composites. We have confirmed that DS ingots of binary and various ternary-alloyed MoSi_2_/Mo_5_Si_3_ eutectic composites exhibiting a homogeneous and fine script lamellar structure can be obtained by controlling the growth rate during DS processing and by adjusting the amount of ternary additions.[16–19] These DS ingots with a homogeneous script lamellar structure are approximated as single crystals having an orientation relationship of (1$\bar{1}$0)_MoSi2_ // (001)_Mo5Si3_ and [110]_MoSi2_ // [1$\bar{1}$0]_Mo5Si3_ with a growth direction being nearly parallel to [1$\bar{1}$0]_MoSi2_ and [001]_Mo5Si3_.[15–17] Our preliminary study on mechanical properties of binary DS eutectic composites suggests that high-temperature strength depends on the average thickness of the MoSi_2_ phase.[20] We also expect that the strength of DS MoSi_2_/Mo_5_Si_3_ eutectic composites depends on the crystal orientation in view of the anisotropic deformation behavior of the tetragonal MoSi_2_ and Mo_5_Si_3_ phases.[7,10,21–26] It is technologically very important to find out the strongest orientation of the DS eutectic composites as well as the most beneficial microstructure (lamellar thickness) of the DS eutectic composites for better high-temperature mechanical properties.

In the present study, we investigate the deformation behavior of DS MoSi_2_/Mo_5_Si_3_ eutectic composites of binary and some ternary alloys as a function of temperature and loading axis orientation in order to elucidate effects of the lamellar spacing and ternary addition on their mechanical properties and the anisotropy in high-temperature strength with the possible causes.

## 2. Experimental procedures

Rod ingots of MoSi_2_/Mo_5_Si_3_ two-phase eutectic composites with nominal compositions of Mo - 54 at.% Si (binary), Mo - 54 at.% Si - 2 at.% X (X = V, Nb, W) and Mo - 54 at.% Si - 5 at.% Ta were prepared by arc-melting. DS ingots of the two-phase eutectic composites were grown from these rod ingots using an optical floating zone furnace at various growth rates between 5 and 100 mm h^–1^ under an Ar gas flow. Microstructures of as-grown DS ingots were examined by scanning electron microscopy (SEM) and transmission electron microscopy (TEM) with JSM-7001FA and JEM-2000FX electron microscopes (JEOL Ltd., Tokyo, Japan), respectively. Chemical compositions were analyzed by energy dispersive X-ray spectroscopy (EDS) in SEM.

Specimens for compression tests with dimensions of 1.2 × 1.2 × 3.0 mm^3^ were cut from the DS ingots by electrical discharge machining (EDM). Compression tests were carried out on an Instron-type testing machine at a strain rate of 1 × 10^−4^ s^−1^ at temperatures ranging from 900 to 1500 °C in vacuum. The loading axis orientations tested are [1$\bar{1}$0]_MoSi2_ and [001]_MoSi2_, which are nearly parallel to and perpendicular to the growth direction of the DS ingots, respectively.[17] Crystallographic orientations of these DS ingots are hereafter referred to those of the MoSi_2_ matrix and are indicated with a subscript ‘MoSi_2_’ unless otherwise stated.

Deformation microstructures were examined by optical microscopy (OM) and TEM. Specimens for TEM observations were sliced from deformed specimens by EDM, mechanically polished and then Ar ion-milled using a JEOL EM-09100IS ion milling machine operated at 6.0 kV for milling and 2.0 kV for finishing.

## 3. Results

### 3.1. Microstructures of the DS ingots

**Figure 1.**
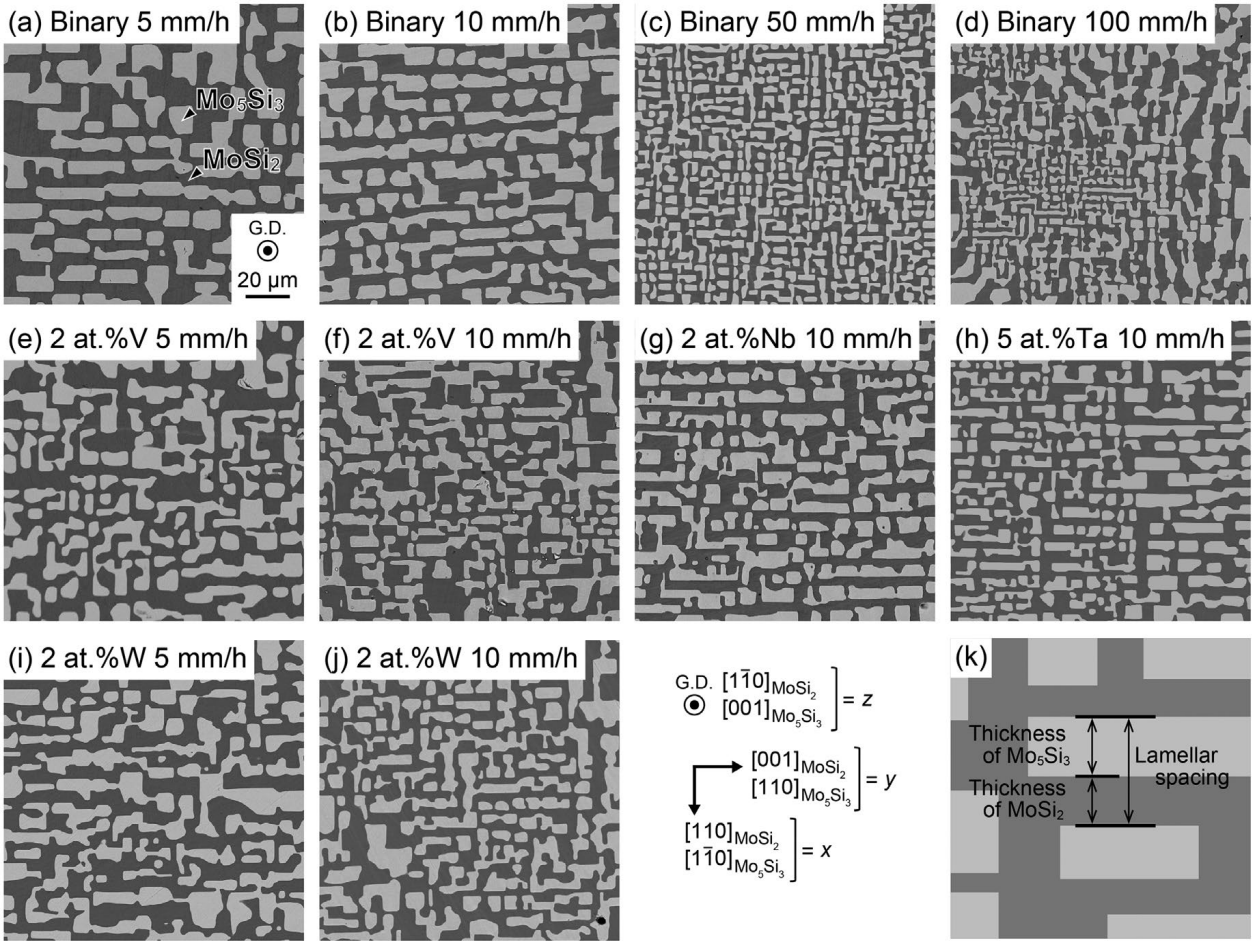
SEM backscattered electron images of DS ingots of (a) Binary 5 mm h^-1^, (b) Binary 10 mm h^-1^, (c) Binary 50 mm h^-1^, (d) Binary 100 mm h^-1^, (e) 2 at.%V 5 mm h^-1^, (f) 2 at.%V 10 mm h^-1^, (g) 2 at.%Nb 10 mm h^-1^, (h) 5 at.%Ta 10 mm h^-1^, (i) 2 at.%W 5 mm h^-1^, (j) 2 at.%W 10 mm h^-1^, (k) Schematic illustration of thickness of each phase and lamellar spacing.

**Figure 2.**
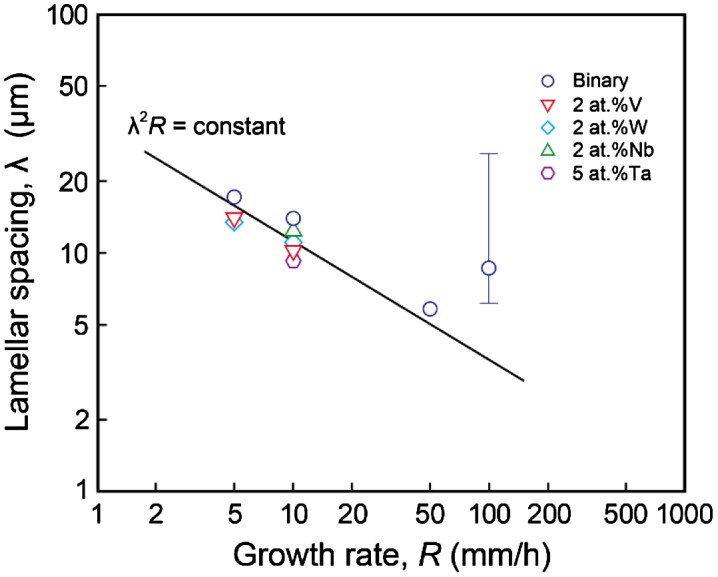
Average lamellar spacing *λ* of binary and some ternary DS eutectic composites plotted as a function of growth rate *R*.

SEM backscattered electron images of a cross-section ((1$\bar{1}$0)_MoSi2_ // (001)_Mo5Si3_) of DS ingots of binary and some ternary eutectic composites grown at various growth rates are shown in Figure 1. Dark and bright regions in the SEM images correspond to MoSi_2_ and Mo_5_Si_3_, respectively. A homogeneous script lamellar structure is observed for most of the DS ingots investigated in this study, except for the binary DS ingot grown at 100 mm h^–1^, in which a cellular structure composed of fine and coarse script lamellar structures is observed. The MoSi_2_ and Mo_5_Si_3_ phases in both homogenous script lamellar and cellular structures are grown nearly along [1$\bar{1}$0]_MoSi2_ and [001]_Mo5Si3_, maintaining the orientation relationship of [110]_MoSi2_ // [1$\bar{1}$0]_Mo5Si3_ and (1$\bar{1}$0)_MoSi2_ // (001)_Mo5Si3_ with a slight deviation of ~2° as previously reported.[15,17] Microstructural characteristics of these DS ingots such as the average thickness and chemical compositions of the MoSi_2_ and Mo_5_Si_3_ phases are summarized in Table 1.^1^ The thickness of each phase was measured along two orthogonal directions, namely [001]_MoSi2_ and [110]_MoSi2_, using test grids drawn on SEM images of the (1$\bar{1}$0)_MoSi2_ cross-sections, as seen in the right bottom of Figure 1. The average lamellar spacing *λ*, which is estimated as a sum of the average thickness of both phases, is plotted in Figure 2 as a function of growth rate. The *λ* value decreases with the increase in the growth rate *R*, following the relationship of *λ* ^2^ *R* = constant, as proposed by Jackson and Hunt [27].

**Table 1.** Average thickness, lamellar spacing and chemical compositions of the MoSi_2_ and Mo_5_Si_3_ phases in binary and some ternary DS eutectic composites grown at various growth rates.

	Growth rate (mm h^–1^)	Average thickness (μm)		Average lamellar spacing (μm)	Chemical composition (at.%)					
		MoSi_2_	Mo_5_Si_3_		MoSi_2_			Mo_5_Si_3_		
					Mo	Si	X	Mo	Si	X
Binary	5	9 ± 8	9 ± 6	18	35.2	64.8	－	63.2	36.8	－
	10	7 ± 6	7 ± 4	14						
	50	3 ± 2	3 ± 1	6						
	100	4 ± 3	4 ± 2	8						
	(cell center)	3 ± 2	3 ± 2	6						
	(cell boundary)	13 ± 10	13 ± 8	26						
2 at.%V	5	8 ± 7	6 ± 4	14	35.2	64.6	0.2	58.7	37.1	4.2
	10	5 ± 4	5 ± 4	10						
2 at.%Nb	10	6 ± 5	6 ± 6	12	36.5	62.5	1.1	57.7	37.2	5.1
5 at.%Ta	10	4 ± 4	5 ± 4	9	34.7	64.5	0.9	47.9	42.0	10.1^1^
2 at.%W	5	7 ± 6	6 ± 4	13	35.1	63.7	1.2	60.4	36.0	3.7^1^
	10	5 ± 4	6 ± 4	11						

**Figure 3.**
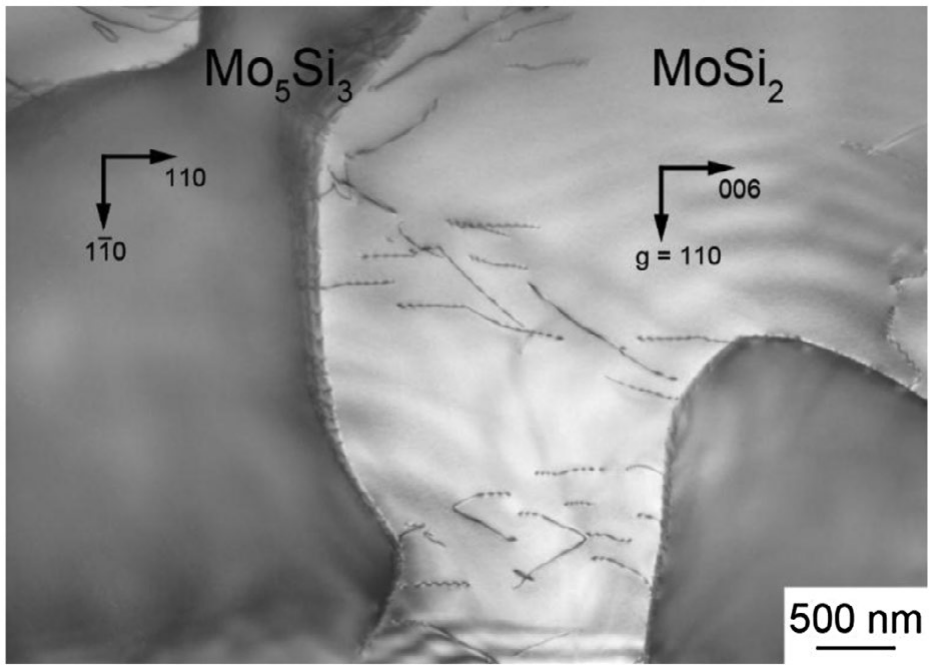
Grown-in dislocations in a binary DS eutectic composites grown at a rate of 100 mm h^–1^.

Figure 3 shows a typical TEM micrograph of a binary DS composites grown at a growth rate of 100 mm h^–1^. The thin foil was cut parallel to the (1$\bar{1}$0)_MoSi2_ // (001)_Mo5Si3_ cross-section. Many grown-in dislocations are observed to exist in the MoSi_2_ matrix but the density of grown-in dislocations in the Mo_5_Si_3_ phase is by far smaller. The Burgers vectors of grown-in dislocations in the MoSi_2_ matrix are confirmed to be <100> by the contrast analysis in TEM. These dislocations are believed to be introduced during cooling by the mismatch of the coefficient of thermal expansion between the two phases. Similar TEM microstructures are observed for all DS ingots.

### 3.2. Stress–strain behavior and yield stress

#### [1$\bar{1}$0]_MoSi2_ orientation

3.2.1. 

**Figure 4.**
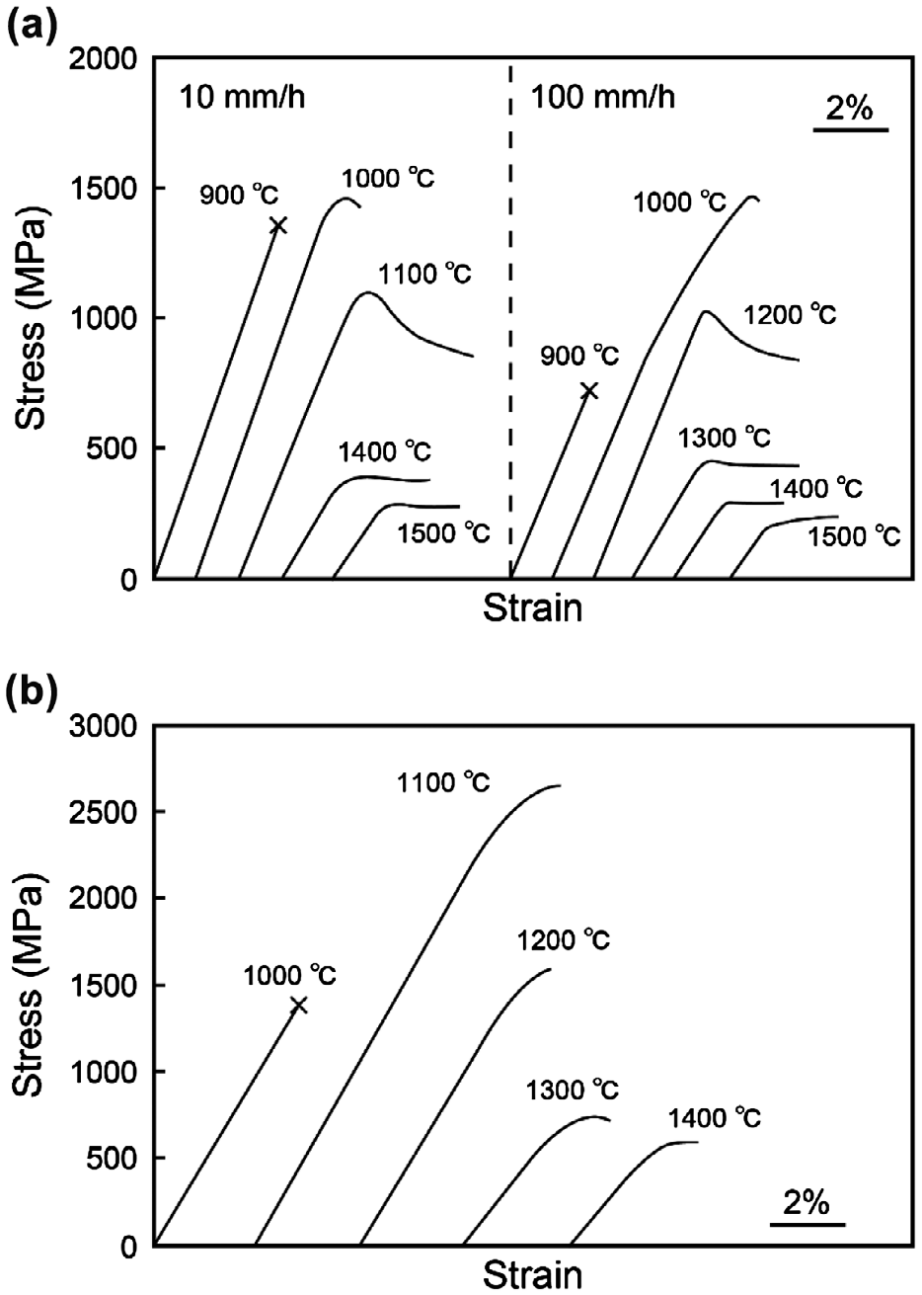
Stress–strain curves of binary DS eutectic composites tested in compression along (a) [1$\bar{1}$0]_MoSi2_ and (b) [001]_MoSi2_.

Typical stress–strain curves for [1$\bar{1}$0]_MoSi2_-oriented specimens of binary DS eutectic composites grown at growth rates of 10 and 100 mm h^–1^ are shown in Figure 4(a). For both specimens, plastic flow is observed only above 1000 °C and premature failure occurs in the elastic region without any appreciable plastic strain below 900 °C. The stress–strain curves for specimens tested between 1000 and 1200 °C generally exhibit a yield drop followed by steady state flow, whereas those above 1300 °C exhibit only nearly steady-state flow from the relatively early stage of plastic deformation.

**Figure 5.**
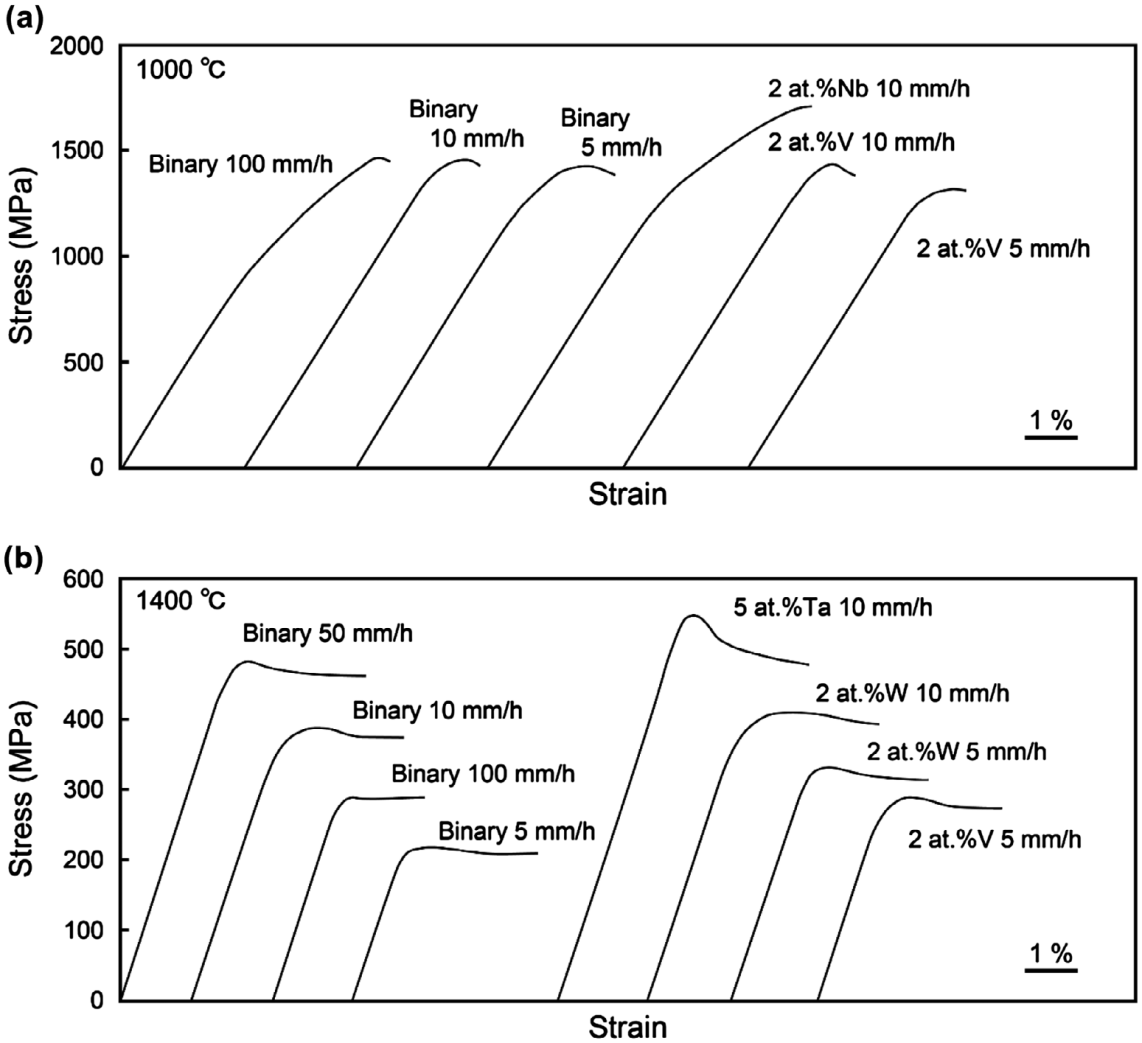
Stress–strain curves obtained for [1$\bar{1}$0]_MoSi2_-oriented specimens of binary and some ternary DS eutectic composites tested at (a) 1000 and (b) 1400 °C.

The variations of stress–strain curves with temperature are similar for [1$\bar{1}$0]_MoSi2_-oriented specimens of all of binary and ternary DS eutectic composites. Typical stress–strain curves obtained for [1$\bar{1}$0]_MoSi2_-oriented specimens of binary and some ternary DS eutectic composites tested at 1000 and 1400 °C are shown in Figure 5. For binary specimens deformed at 1000 °C, the yield stress (flow stress at 0.2% plastic strain) depends on the growth rate, while the maximum stress reached before the yield drop is almost independent of the growth rate. A similar trend is also observed for V-alloyed specimens. At 1400 °C, in contrast, both the yield stress and maximum stress depend on the growth rate for the binary and W-alloyed specimens. The difference in temperature variations of stress–strain curves between 1000 and 1400 °C is ascribed to the difference in the deformation mechanisms, as described later in the next section on deformation microstructure observations.

**Figure 6.**
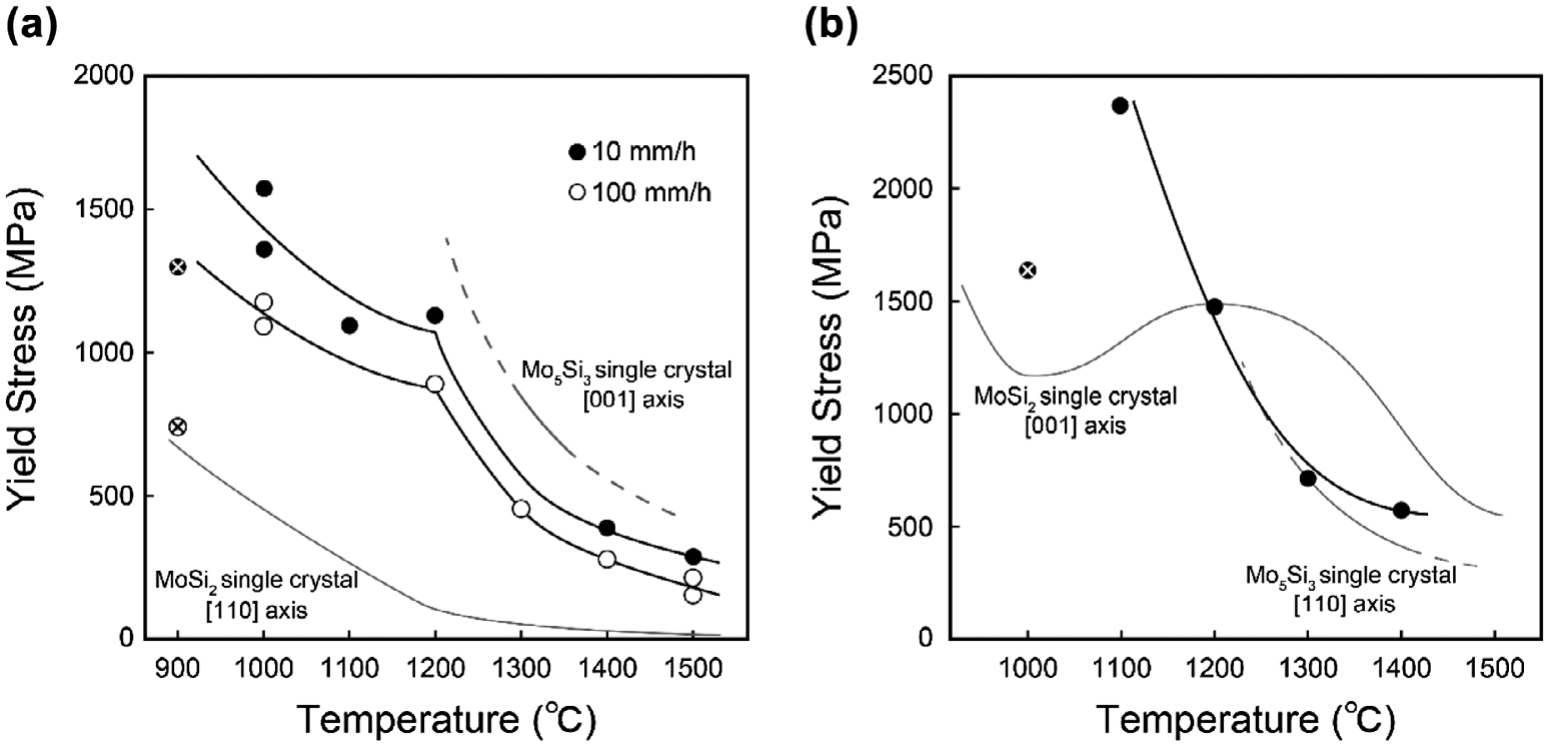
Temperature dependence of yield stress for (a) [1$\bar{1}$0]_MoSi2_ and (b) [001]_MoSi2_-oriented specimens of binary DS eutectic composites. Marks × in open and filled circles indicate stresses at which failure occurs without any plastic flow.

Figure 6 shows the temperature dependence of yield stress of [1$\bar{1}$0]_MoSi2_-oriented specimens of binary DS eutectic composites grown at growth rates of 10 mm h^–1^ (homogeneous script lamellar structure) and 100 mm h^–1^ (cellular structure), together with those of single crystals of MoSi_2_ and Mo_5_Si_3_ with [1$\bar{1}$0]_MoSi2_ and [001]_Mo5Si3_ orientations, respectively, for references [24–26]. Values of yield stress for both specimens of binary DS eutectic composites decreases with increasing temperature and are several times higher than those of MoSi_2_ single crystals with the corresponding orientation at all temperatures. The yield stress is higher for specimens with a homogeneous script lamellar structure (*R* = 10 mm h^–1^) than for specimens with cellular structures (*R* = 100 mm h^–1^) at all temperatures, indicating the importance of the formation of a homogeneous and fine script lamellar structure for better high-temperature strength of DS MoSi_2_/Mo_5_Si_3_ eutectic composites. Of importance to note in Figure 6(a) is that the yield stress for the DS MoSi_2_/Mo_5_Si_3_ eutectic composite with a fine homogeneous script lamellar structure (*R* = 10 mm h^–1^) is very high at high temperatures, exceeding 385 MPa at 1400 °C and 290 MPa at 1500 °C.

#### 3.2.2. [001]_MoSi2_ orientation

Figure 4(b) shows typical stress–strain curves for [001]_MoSi2_-oriented binary DS composite specimens grown at *R* = 10 mm h^–1^. Plastic flow is observed only above 1100 °C, which is 100 °C higher than the onset temperature for [1$\bar{1}$0]_MoSi2_-oriented binary DS composite specimens. The yield stress decreases drastically and monotonously with increasing temperature, as shown in Figure 6(b), in which the temperature dependence of yield stress is also shown for single crystals of MoSi_2_ with the [001]_MoSi2_ orientation for reference [24]. Although the value of yield stress of the binary DS composite specimen is much higher than that of the [001]-oriented MoSi_2_ single crystal at 1100 °C, the opposite is true at and above 1300 °C.

This implies that the strength of single crystals of Mo_5_Si_3_ is lower than that of single crystals of MoSi_2_ for the corresponding orientations at and above 1300 °C. Our preliminary results on compression tests of Mo_5_Si_3_ single crystals with the corresponding and [110]_Mo5Si3_ loading axis orientation indeed indicate that the values of yield stress of [110]_Mo5Si3_-oriented Mo_5_Si_3_ single crystals at 1300 and 1400 °C are close to and a little lower than those of [001]_MoSi2_-oriented DS eutectic composites, respectively.[28] This implies the importance of the increase of strength of the Mo_5_Si_3_ phase for obtaining better high-temperature strength of [001]_MoSi2_-oriented DS MoSi_2_/Mo_5_Si_3_ eutectic composites at high temperatures above 1300 °C. It is worth noting in Figure 6(b) that the yield stress for the DS MoSi_2_/Mo_5_Si_3_ eutectic composite with a fine homogeneous script lamellar structure (*R* = 10 mm h^–1^) is very high, exceeding 500 MPa at 1400 °C, which is by far higher than any other values reported so far for high-temperature materials.

### 3.3. Deformation microstructures

#### [1$\bar{1}$0]_MoSi2_ orientation

3.3.1. 

**Figure 7.**
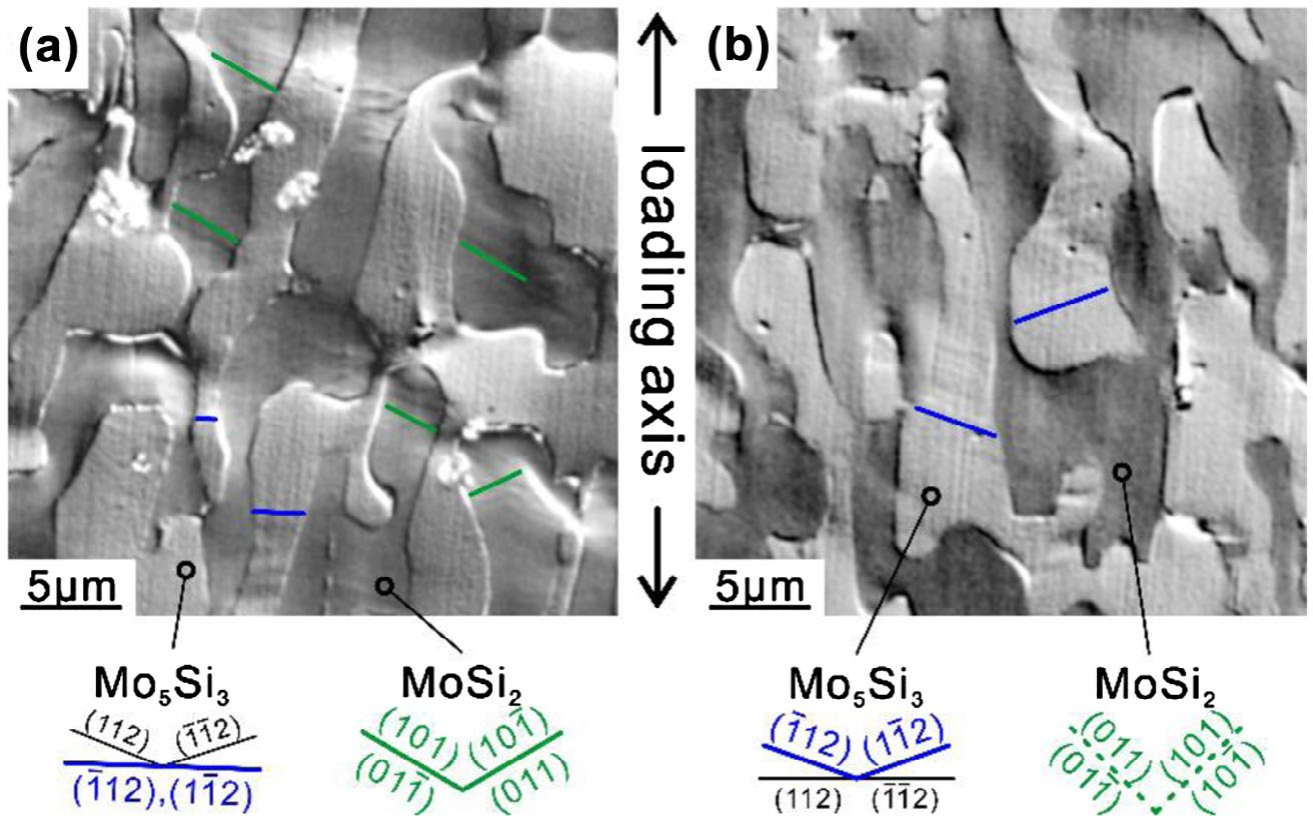
Deformation markings observed on two orthogonal surfaces parallel to (a) (110)_MoSi2_ and (b) (001)_MoSi2_ of a [1$\bar{1}$0]_MoSi2_-oriented specimen of a binary DS eutectic composite deformed at 1000 °C.

Deformation markings observed on two orthogonal surfaces of a [1$\bar{1}$0]_MoSi2_-oriented specimen of the binary DS composite deformed to ~1% plastic strain at 1000°C are shown in Figure 7. Fine deformation markings are observed in the MoSi_2_ matrix parallel to the traces of {011} planes on the (110)_MoSi2_ surface (Figure 7(a)) while those observed in the MoSi_2_ matrix on the (001)_MoSi2_ surface are very faint (Figure 7(b)), indicating that the slip directions of the MoSi_2_ matrix are contained on the (001)_MoSi2_ surface. The slip directions are thus inferred to be [100] and [010], which is confirmed by TEM analysis of dislocations introduced during deformation as described later in this section. The slip system activated in the MoSi_2_ matrix is thus determined to be {011}<100>. Deformation markings observed in the Mo_5_Si_3_ phase are coarse on the (110) _MoSi2_ // (1$\bar{1}$0)_Mo5Si3_ surface (Figure 7(a)), while those in the Mo_5_Si_3_ phase are again very faint on the (001)_MoSi2_ // (110)_Mo5Si3_ surface (Figure 7(b)). When judged from the appearance of deformation markings on these two surfaces, slip planes of the Mo_5_Si_3_ phase are determined to be (1$\bar{1}$2) and ($\bar{1}$12), two of the possible four {112} planes. Since the shortest Burgers vector on {11$\bar{2}$} is 1/2<111>, the slip system of {11$\bar{2}$}<111> is expected to be activated in Mo_5_Si_3_ at 1000 °C.

**Figure 8.**
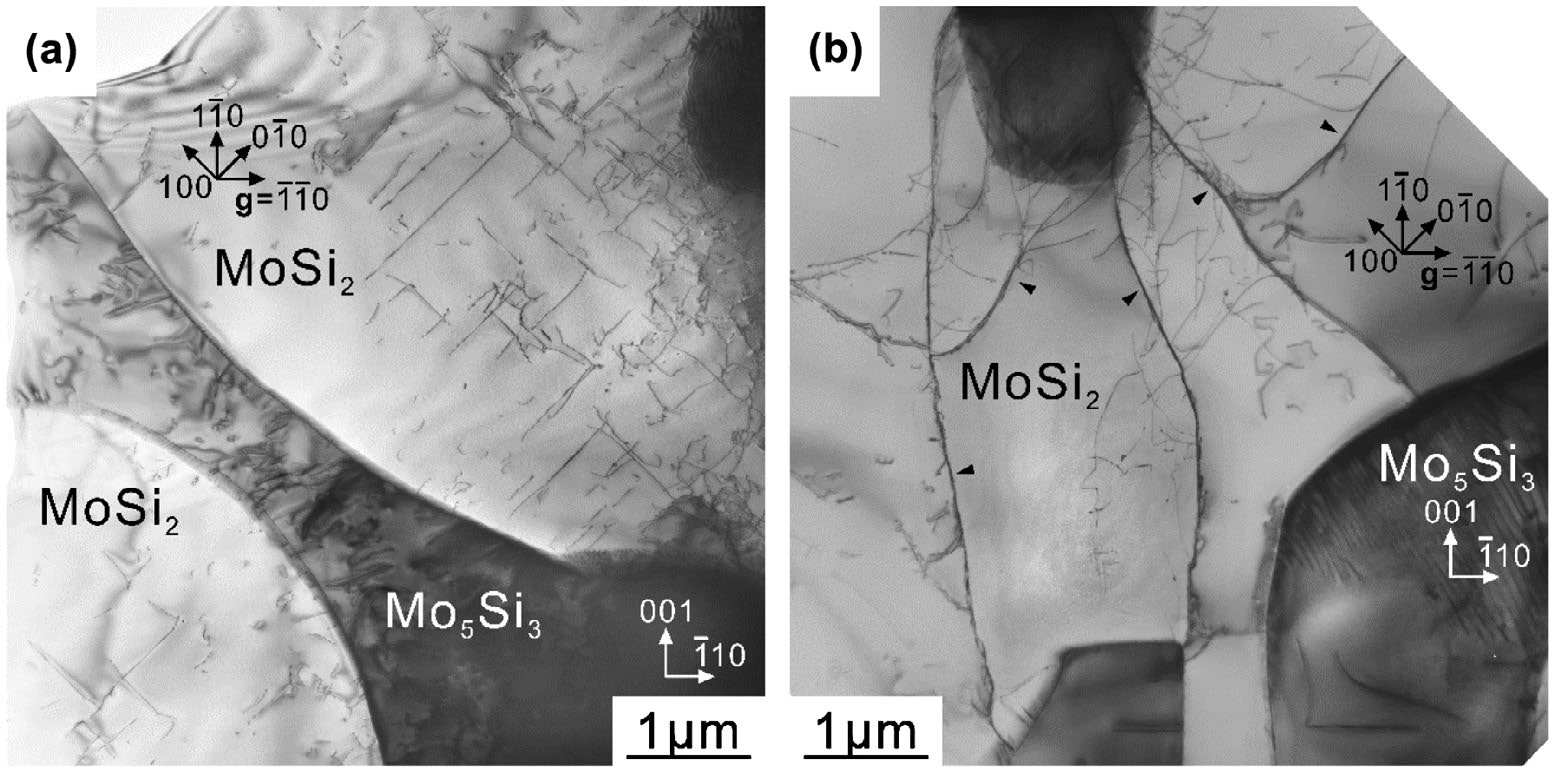
TEM bright-field images of [1$\bar{1}$0]_MoSi2_-oriented specimens of binary DS eutectic composites deformed at (a) 1000 °C and (b) 1400 °C.

A TEM bright-field (BF) image of a [1$\bar{1}$0]_MoSi2_-oriented binary DS composite specimen deformed to ~1% plastic strain at 1000 °C is shown in Figure 8(a). Many dislocations are observed in both MoSi_2_ and Mo_5_Si_3_, indicating that dislocations are activated in both phases at 1000 °C. The Burgers vectors of dislocations in the MoSi_2_ matrix are confirmed to be [100] and [010] (hereafter designated as **b**_1_ and **b**_2_), which are identical to those for as-grown dislocations (Figure 3). The activation of <100> dislocations are similarly observed in compression of [1$\bar{1}$0]_MoSi2_-oriented MoSi_2_ single crystals.[7] Since plastic flow is observed only above 1300 °C for Mo_5_Si_3_ single crystals with the [001] orientation,[25,26] the introduction of dislocations into the Mo_5_Si_3_ phase in the script lamellar structure is considered to be assisted by the stress concentration generated by dislocation pile-ups against the interphase boundary in the MoSi_2_ matrix. Then, it is reasonable to consider that plastic deformation is initiated in the MoSi_2_ matrix and that it propagates into Mo_5_Si_3_ after the stress concentration at the interphase boundary due to pile-up dislocations in the MoSi_2_ matrix reaches some critical values. As seen in Figure 5, for binary specimens deformed at 1000 °C, the yield stress (flow stress at 0.2% plastic strain) depends on the growth rate, while the maximum stress reached before the yield drop is almost independent of the growth rate. The yield stress may correspond to the stress level at which plastic deformation is initiated in the MoSi_2_ matrix, while the maximum stress primarily to the stress level at which plastic deformation starts to propagate into the Mo_5_Si_3_ phase.

**Figure 9.**
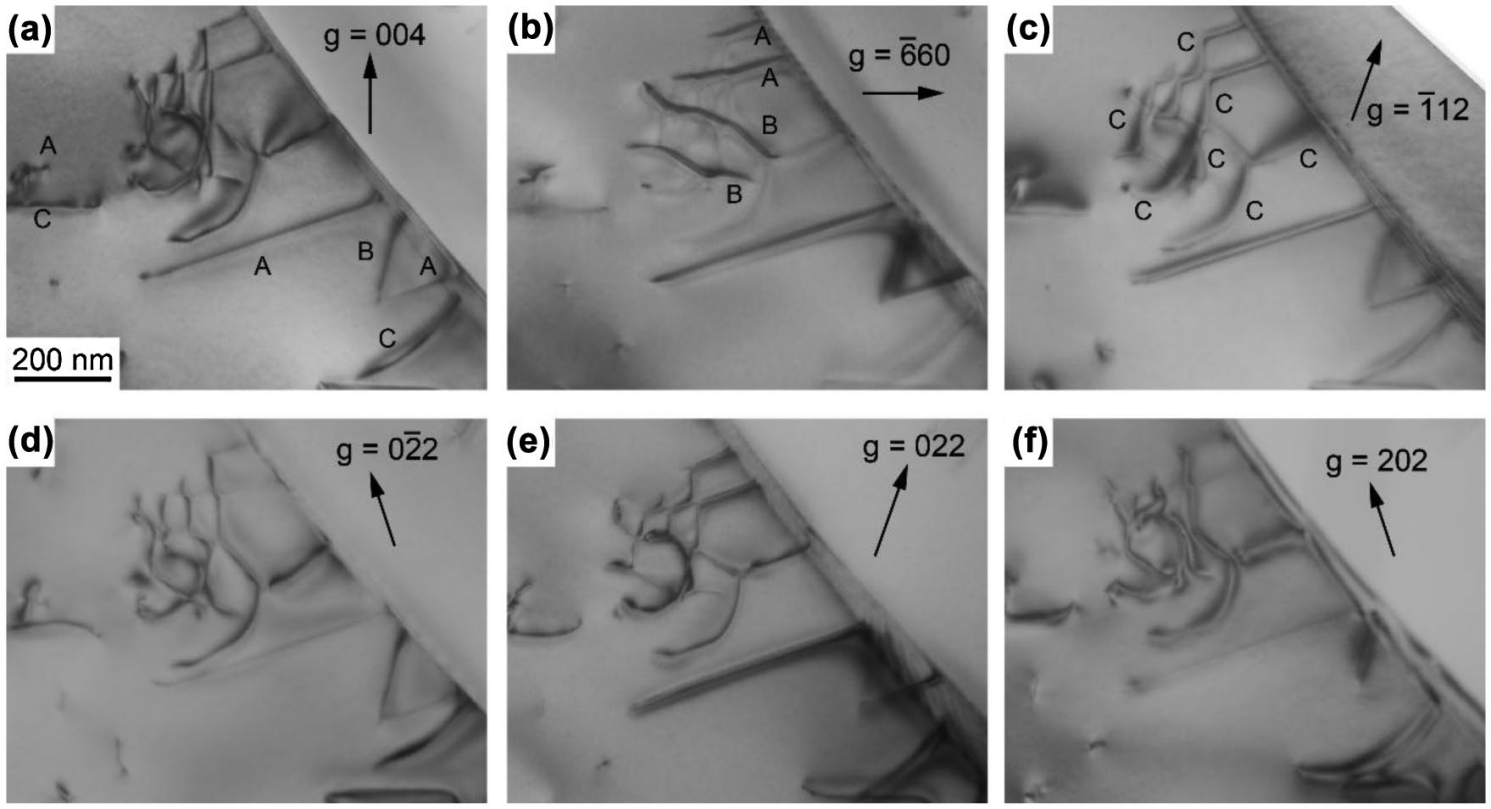
Contrast analysis of dislocations in Mo_5_Si_3_ in a [1$\bar{1}$0]_MoSi2_-oriented specimen of a binary DS eutectic composite deformed at 1000 °C. The diffraction vector (g) used is indicated in each of the images.

Figure 9 shows a result of contrast analysis made for dislocations introduced in the Mo_5_Si_3_ phase at 1000 °C. Dislocations having three different Burgers vectors as marked A, B and C exist in Mo_5_Si_3_. Dislocations marked A are invisible when the diffraction vectors (**g**) are 0$\bar{2}$2 (Figure 9(d)) and 202 (Figure 9(f)). The Burgers vector (**b**_A_) of the dislocation A is thus determined to be parallel to [1$\bar{1}$ $\bar{1}$] according to the standard **g•b** rule. The Burgers vector (**b**_B_) of dislocations marked B is identified to be parallel to [1$\bar{1}$1] because it is invisible for **g** = $\bar{1}$12 (Figure 9(c)) and **g** = 022 (Figure 9(e)). For Mo_5_Si_3_ with a body-centered tetragonal crystal structure of the D8 _m_ type (space group: *I*4/*mcm*), the shortest translation vector along <111> is 1/2<111>. The Burgers vectors of the observed dislocations A and B are thus inferred to be 1/2[1$\bar{1}$ $\bar{1}$] and 1/2[1$\bar{1}$1], respectively. These observations are consistent with the result of trace analysis shown in Figure 7. Dislocations marked C are invisible for **g** = $\bar{3}$30 (Figure 9(b)), while they are visible for the other imaging conditions presented in Figure 9. Among possible Burgers vectors with relatively short magnitudes, i.e. [001], 1/2<111>, <100> and <011>, only **b** = [001] can satisfy the visibility conditions. The Burgers vector (**b**_C_) of the dislocation C is thus determined to be [001]. Under the assumption that in DS MoSi_2_/Mo_5_Si_3_ eutectic composites with the corresponding orientation, plastic deformation initiates in the MoSi_2_ matrix and propagates into the Mo_5_Si_3_ phase across the interphase boundary, the observed dislocations (**b**_1_, **b**_2_ in MoSi_2_, **b**_A_, **b**_B_, **b**_C_ in Mo_5_Si_3_) can be correlated with each other as follows.

(1) $$\begin{array}{c} 30\times b_{1}\approx 10\times b_{\text{B}}+\;9\times b_{\text{C}}\\ 30\times b_{2}\approx 10\times b_{\text{A}}+\;9\times \left(-b_{\text{C}}\right) \end{array}$$

In Mo_5_Si_3_, while the dislocations A and B with **b** = 1/2<111>(**b**_A_ and **b**_B_) can glide on their relevant slip planes under external stress, the dislocation C (**b**_C_ = [001]) cannot glide so easily because of no shear stress acting on it. The dislocation C is thus expected to exist only in the vicinity of the interphase boundary and sometimes to annihilate by a reaction with the dislocation of opposite sign (**b**_C_ and −**b**_C_). The slip planes for the dislocations A and B (**b**_A_ and **b**_B_) are (1$\bar{1}$2) and ($\bar{1}$12). This is consistent with the result of slip trace analysis of Figure 7 that the slip traces observed are only those corresponding to slip on (1$\bar{1}$2) and ($\bar{1}$12), two of the possible four {112} planes.

Figure 8(b) shows a TEM BF image of a [1$\bar{1}$0]_MoSi2_-oriented specimen of the binary DS composite compressed to ~2% plastic strain at 1400 °C. In the MoSi_2_ matrix, a high density of long and curved <100> dislocations mostly with non-screw characters frequently form dislocation nodes and sub-boundaries (some are marked with arrowheads in Figure 8(b)), indicating the occurrence of dislocation climb in the MoSi_2_ matrix at about 2% plastic strain corresponding to a nearly steady-state flow. The dislocation structure in the Mo_5_Si_3_ phase does not differ much from that observed in the specimen deformed at 1000 °C. Although the activation of dislocations in both MoSi_2_ and Mo_5_Si_3_ phases are observed, the dislocation density is much higher in MoSi_2_ than in Mo_5_Si_3_, suggesting that plastic deformation occurs dominantly in the MoSi_2_ matrix. This may be the reason why both the yield stress and maximum stress vary with the growth rate in marked contrast to the case of 1000 °C.

#### 3.3.2. [001]_MoSi2_ loading axis

**Figure 10.**
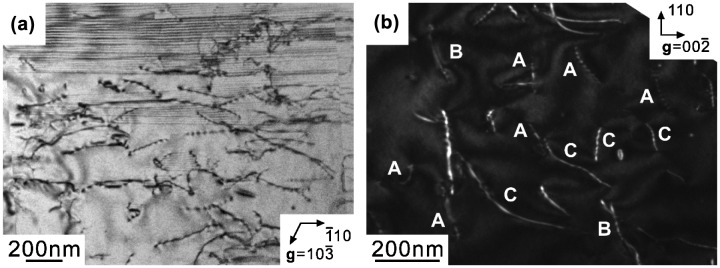
Deformation microstructures of (a) MoSi_2_ and (b) Mo_5_Si_3_ in a [001]_MoSi2_-oriented specimen of a binary DS eutectic composite deformed at 1100 °C.

Figure 10 shows deformation microstructures of MoSi_2_ and Mo_5_Si_3_ phases in a [001]_MoSi2_-oriented binary DS composite specimen deformed at 1100 °C. A high density of perfect dislocations and stacking faults on (001) are observed in the MoSi_2_ matrix. The Burgers vectors of the perfect dislocations and the partial dislocations bounding the stacking fault in Figure 10(a) are determined to be parallel to [100] and mostly <331>, respectively. Since no resolved shear stress acts along [100] with the [001] loading axis direction, the [100] dislocation cannot carry plastic strain by simple glide on any of their possible slip planes such as {010}, {011}, {023}, {013} and {001}. In addition, the (001) stacking fault plane cannot be a slip plane for partial dislocations with the Burgers vector parallel to <331>. Stacking faults on (001) in as-grown and plastically deformed MoSi_2_ have been observed by many researchers.[22,23,29–31]. We have also observed similar stacking faults on (001) formed during high-temperature compression tests of [001]-oriented single crystals of WSi_2_ with the C11_b_ crystal structure and proposed a possible formation mechanism, in which the (001) stacking fault is formed by the climb motion of 1/6<331> partial dislocations that originate from a <100> perfect dislocation.[32]

Although the formation mechanism of stacking faults on (001) observed in the MoSi_2_ matrix of the present DS eutectic composite specimens has yet to be clarified, it is reasonable to consider that the formation of these (001) stacking faults during deformation at 1100 °C (~0.6 T_m_, T_m_: melting point) involves a significant diffusion process similarly to the case proposed for WSi_2_ single crystals.[32]

In Mo_5_Si_3_, dislocations with three different Burgers vectors marked A, B and C are determined to have Burgers vectors of 1/2[111], 1/2[11$\bar{1}$] and [001] respectively by contrast analysis. Since no shear stress acts along [001] with the [110]_Mo5Si3_ loading axis orientation, the dislocation C (**b** = [001]) is considered to be introduced as a result of dislocation reactions between two 1/2<111>.

## 4. Discussion

### 4.1. Deformation modes and strain compatibility

If a crystal is sheared by a small amount *s* by the operation of a slip system whose slip direction and slip plane normal are respectively parallel to the *x*_0_ and *y*_0_ axes of the (*x*_0_, *y*_0_, *z*_0_) orthogonal coordinate system, the strain tensor *ε*_0*ij*_ of the shear deformation is given by the following equation:[33,34]

(2) $$\varepsilon_{0\text{ij}}\;\;\text{=}\;\left[\begin{array}{ccc} 0 & s\text{/2} & 0\\ s\text{/2} & 0 & 0\\ 0 & 0 & 0 \end{array}\right]$$

In order to discuss the strain compatibility between the two constituent phases in DS MoSi_2_/Mo_5_Si_3_ eutectic composites, the strain tensor for the relevant operative slip systems has to be transformed so as to be described with the (*x*, *y*, *z*) orthogonal coordinate system for the MoSi_2_/Mo_5_Si_3_ eutectic composite, where *x* = [110]_MoSi2_ // [1$\bar{1}$0]_Mo5Si3_, *y* = [001]_MoSi2_ // [110]_Mo5Si3_, *z* = [1$\bar{1}$0]_MoSi2_ // [001]_Mo5Si3_. A slight misalignment of about 2° between [1$\bar{1}$0]_MoSi2_ and [001]_Mo5Si3_ around the *x* axis is neglected here for simplicity, since the strain components are hardly affected by the misalignment.

In [1$\bar{1}$0]_MoSi2_-oriented DS MoSi_2_/Mo_5_Si_3_ eutectic composites deformed at and above 1000 °C, the dominant deformation mode observed is {011}<100> slip in MoSi_2_ and is {11$\bar{2}$}<111> slip in Mo_5_Si_3_. Strain components calculated for these slip systems in MoSi_2_ and Mo_5_Si_3_ are summarized in Tables 2 and 3, respectively. The strain components in Tables 2 and 3 are normalized to their respective absolute value of *ε*_*zz*_. The strain component *ε*_*zz*_ corresponds to the normal strain along the compression axis since the loading axis is parallel to the *z* axis in this case. Under the condition of uniaxial loading along [1$\bar{1}$0]_MoSi2_, four {011}<100> equivalent slip systems are equally stressed with the identical Schmid factors of 0.463 and these four slip systems are assumed to be equally activated in MoSi_2_. The strain tensor for the MoSi_2_ matrix is then given in the following form.

(3) $$\varepsilon_{ij}\;\;\text{=}\;\left[\begin{array}{ccc} \varepsilon_{xx} & 0 & 0\\ 0 & 0 & 0\\ 0 & 0 & \varepsilon_{zz} \end{array}\right]$$

**Table 2.** Strain components for {011}<100> slip systems in the MoSi_2_ phase calculated with respect to the (*x*, *y*, *z*) orthogonal coordinate system for the MoSi_2_/Mo_5_Si_3_ eutectic composite, where *x* = [110]_MoSi2_, *y* = [001]_MoSi2_, *z* = [1$\bar{1}$0]_MoSi2_. Schmid factors for uniaxial loading along *z* = [1$\bar{1}$0]_MoSi2_ are also indicated.

Slip system	*ε*_xx_	*ε*_yy_	*ε*_zz_	*ε*_yz_	*ε*_zx_	*ε*_xy_	Schmid factor for *z*-axis compression
(011)[100]	1.000	0.000	−1.000	0.289	0.000	0.289	0.463
(01$\bar{1}$)[100]	1.000	0.000	−1.000	−0.289	0.000	−0.289	0.463
(101)[010]	1.000	0.000	−1.000	−0.289	0.000	0.289	0.463
(10$\bar{1}$)[010]	1.000	0.000	−1.000	0.289	0.000	−0.289	0.463

**Table 3.** Strain components for {11$\bar{2}$}<111> slip systems in the Mo_5_Si_3_ phase calculated with respect to the (*x*, *y*, *z*) orthogonal coordinate system for the MoSi_2_/Mo_5_Si_3_ eutectic composite, where *x* =[1$\bar{1}$0]_Mo5Si3_, *y* =[110]_Mo5Si3_, *z* = [001]_Mo5Si3_. Schmid factors for uniaxial loading along *z* = [001]_Mo5Si3_ and *y* = [110]_Mo5Si3_ are also indicated.

Slip system	*ε*_xx_	*ε*_yy_	*ε*_zz_	*ε*_yz_	*ε*_zx_	*ε*_xy_	Schmid factor for *z*-axis compression	Schmid factor for *y*-axis compression
(11$\bar{2}$)[111]	0.000	1.000	−1.000	−1.212	0.000	0.000	0.318	0.318
(112)[11$\bar{1}$]	0.000	1.000	−1.000	1.212	0.000	0.000	0.318	0.318
($\bar{1}$1$\bar{2}$)[$\bar{1}$11]	1.000	0.000	−1.000	0.000	1.212	0.000	0.318	0
($\bar{1}$12)[$\bar{1}$1$\bar{1}$]	1.000	0.000	−1.000	0.000	−1.212	0.000	0.318	0

where $\varepsilon_{xx}/\varepsilon_{zz}=-1$. In Mo_5_Si_3_, there are four equivalent {11$\bar{2}$}<111> slip systems with the identical Schmid factor (0.318) under the condition of uniaxial loading along [001]_Mo5Si3_. However, only two slip systems, (1$\bar{1}$2)[1$\bar{1}$ $\bar{1}$] and ($\bar{1}$12)[$\bar{1}$1$\bar{1}$], of the four are confirmed to be operative experimentally in the present study. On the assumption that these two slip systems are equally activated, the strain tensor for Mo_5_Si_3_ is described also by Equation (3). If the plastic strain tensors of the two constituent phases are identical, macroscopic plastic deformation is expected to occur without introducing any cracks at the interphase boundary because no strain incompatibility is developed at the boundary. We believe that this is actually what happened in the deformation of DS MoSi_2_/Mo_5_Si_3_ eutectic composites along [1$\bar{1}$0]_MoSi2_ above 1000 °C. In fact, if the other two {11$\bar{2}$}<111> slip systems, (11$\bar{2}$)[111] and (112)[11$\bar{1}$], of the four are equally activated, the strain tensor is totally different from Equation (3) having non-zero values only for the strain components of $\varepsilon_{yy}^{}$ and $\varepsilon_{zz}^{}$ ($\varepsilon_{zz}^{}/\varepsilon_{yy}^{}=-1$). Since the strain compatibility cannot be achieved at the interphase boundary in this case, plastic deformation will hardly occur in this case. In other words, there is a reason for the preferential activation of (1$\bar{1}$2)[1$\bar{1}$ $\bar{1}$] and ($\bar{1}$12)[$\bar{1}$1$\bar{1}$] slip systems in Mo_5_Si_3_.

In [001]_MoSi2_-oriented DS MoSi_2_/Mo_5_Si_3_ eutectic composites, in contrast, the onset temperature (1100 °C; ~0.6 T_m_) for plastic flow is found to be higher than that for [1$\bar{1}$0]_MoSi2_-oriented specimens. This is because plastic deformation of [001]_MoSi2_-oriented specimens needs the operation of diffusion-controlled deformation processes such as the climb motion of dislocations accompanied by the formation of (001) stacking faults in MoSi_2_. We now consider how plastic deformation of [001]_MoSi2_-oriented DS MoSi_2_/Mo_5_Si_3_ eutectic composites occurs at temperatures lower than the actually observed onset temperature (1100 °C) for plastic flow without diffusion-controlled processes. The activation of {013}<3$\bar{3}$1> slip is expected in MoSi_2_, because it is the operative deformation mode observed in [001]-oriented MoSi_2_ single crystals.[7,21–24]. Strain components generated by the activation of eight equivalent {013}<3$\bar{3}$1> slip systems in MoSi_2_ are summarized in Table 4. The strain component *ε*_*yy*_ corresponds to the normal strain along the compression axis in this case, since the [001]_MoSi2_-loading axis corresponds to the y-axis in the (*x*, *y*, *z*) orthogonal coordinate system. All of the eight equivalent {013}<3$\bar{3}$1> slip systems with the identical Schmid factor (0.387) are expected to be equally operative in the MoSi_2_ matrix under the condition of uniaxial compression along [001] of MoSi_2_. Since the number of independent slip systems for general deformation is sufficiently high, any resultant macroscopic strain can be achieved by simultaneous activation of some of the eight slip systems. For example, if (013)[ $\bar{3}$3$\bar{1}$], (01$\bar{3}$)[ $\bar{3}$31], (103)[3$\bar{3}$ $\bar{1}$] and ($\bar{1}$03)[ $\bar{3}$3$\bar{1}$] slip systems are equally activated, the resultant macroscopic strain tensor is described by the following form,

**Table 4.** Strain components for {10$\bar{3}$}<331> slip systems in the MoSi_2_ phase calculated with respect to the (*x*, *y*, *z*) orthogonal coordinate system for the MoSi_2_/Mo_5_Si_3_ eutectic composite, where *x*  =  [110]_MoSi2_, *y*  =  [001]_MoSi2_, *z* = [1$\bar{1}$0]_MoSi2_. Schmid factors for uniaxial loading along *y* = [001]_MoSi2_ are also indicated.

Slip system	*ε*_xx_	*ε*_yy_	*ε*_zz_	*ε*_yz_	*ε*_zx_	*ε*_xy_	Schmid factor for *y*-axis compression
(103)[3$\bar{3}$$\bar{1}$]	0.000	−1.000	1.000	0.578	0.500	−0.288	0.387
(103)[33$\bar{1}$]	1.000	−1.000	0.000	−0.288	0.500	0.578	0.387
($\bar{1}$03)[$\bar{3}$$\bar{3}$$\bar{1}$]	1.000	−1.000	0.000	0.288	0.500	−0.578	0.387
($\bar{1}$03)[$\bar{3}$3$\bar{1}$]	0.000	−1.000	1.000	−0.578	0.500	0.288	0.387
(013)[$\bar{3}$3$\bar{1}$]	0.000	−1.000	1.000	−0.578	−0.500	−0.288	0.387
(013)[33$\bar{1}$]	1.000	−1.000	0.000	0.288	−0.500	0.578	0.387
(0$\bar{1}$3)[$\bar{3}$$\bar{3}$$\bar{1}$]	1.000	−1.000	0.000	−0.288	−0.500	−0.578	0.387
(0$\bar{1}$3)[3$\bar{3}$$\bar{1}$]	0.000	−1.000	1.000	0.578	−0.500	0.288	0.387

(4) $$\varepsilon_{ij}\;=\;\left[\begin{array}{ccc} 0 & 0 & 0\\ 0 & \varepsilon_{yy} & 0\\ 0 & 0 & \varepsilon_{zz} \end{array}\right]$$　　　

where $\varepsilon_{zz}^{}/\varepsilon_{yy}^{}=-1$. In contrast, (001)<110>slip is only the slip system so far reported in Mo_5_Si_3_.[25,26] However, there is no shear stress acting on this slip system when the compression axis is [110] of Mo_5_Si_3_. We, therefore, assume that {11$\bar{2}$}<111> slip, which is newly identified in the present study, is operative under the condition of uniaxial loading along [110] of Mo_5_Si_3_. Strain components for four equally stressed equivalent {11$\bar{2}$}<111> slip systems are given in Table 3. The strain component *ε*_*yy*_ corresponds to the normal strain along the compression axis in this case, since the [110]_Mo5Si3_-loading axis corresponds to the y-axis in the (*x*, *y*, *z*) orthogonal coordinate system. On the assumption that the ($\bar{1}$ $\bar{1}$2)[111] and (112)[$\bar{1}$ $\bar{1}$1] slip systems with a non-zero Schmid factor (0.318) are operative equally, the resultant macroscopic strain tensor becomes identical to that given by Equation (4). Then, strain compatibility at the interphase boundary is expected to be satisfied, as far as the resultant macroscopic strain tensor described by Equation (4) is achieved in each of the constituent phases. However, no apparent plastic flow is observed in [001]_MoSi2_-oriented DS MoSi_2_/Mo_5_Si_3_ eutectic composites below 1000 °C, indicating that strain compatibility is not actually achieved at the interphase boundary. We believe that the macroscopic strain tensor generated in MoSi_2_ cannot be in the form of Equation (4), which needs the simultaneous activation of some of the eight equivalent {013}<3$\bar{3}$1> slip systems. Indeed, [001]-oriented MoSi_2_ single crystals have been reported to exhibit extremely high work-hardening followed by early fracture at about 0.5% plastic strain below 1200 °C.[24] This clearly suggests that macroscopic flow accompanied by the multiple activation of {013}<3$\bar{3}$1> slip systems in MoSi_2_ so as to satisfy the strain compatibility at the interphase boundary is difficult to achieve in [001]_MoSi2_-oriented DS MoSi_2_/Mo_5_Si_3_ eutectic composites below 1000 °C and that plastic deformation of this orientation occurs only at higher temperatures where diffusion-controlled deformation processes play an important role. In other words, the difficulty in multiple activation of {013}<3$\bar{3}$1> slip systems in the MoSi_2_ matrix is the reason for the higher onset temperature for plastic flow for [001]_MoSi2_-oriented DS MoSi_2_/Mo_5_Si_3_ eutectic composites.

### 4.2. Influences of growth rate and ternary addition on high temperature strength

**Figure 11.**
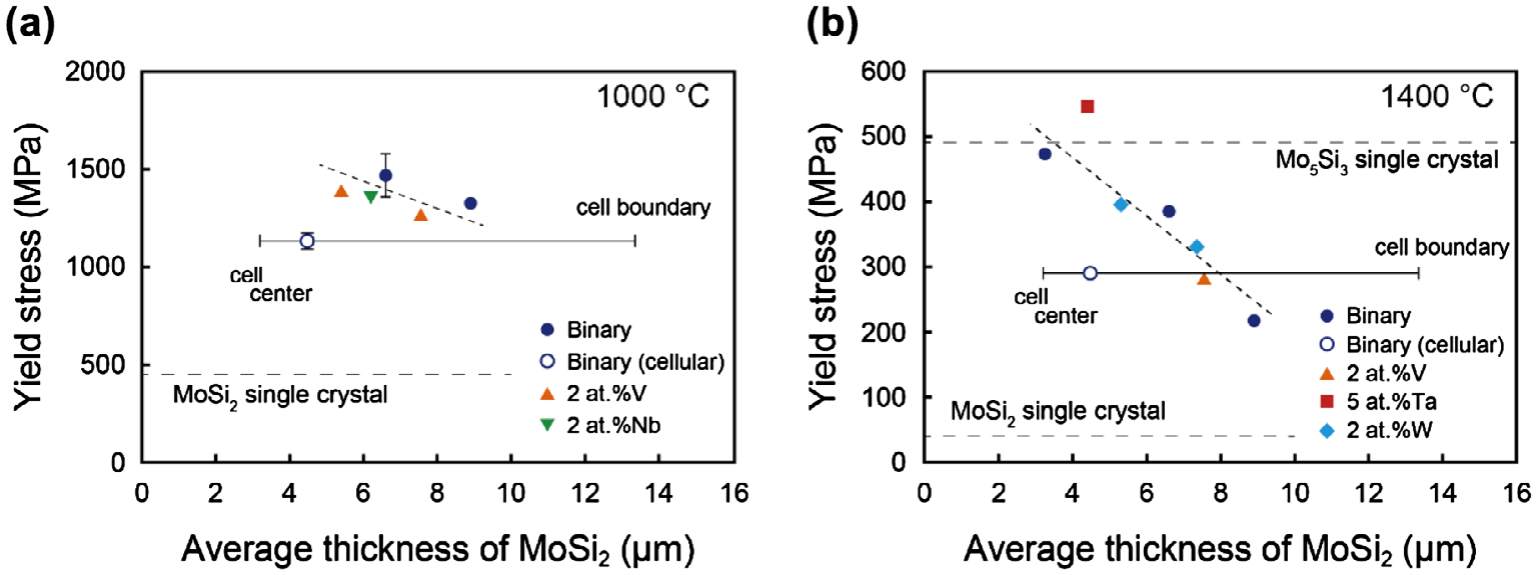
Yield stress of the [1$\bar{1}$0]_MoSi2_-oriented specimens of binary and some ternary DS eutectic composites deformed at (a) 1000 °C and (b) 1400 °C plotted as a function of the average thickness of MoSi_2_.

The yield stress of [1$\bar{1}$0]_MoSi2_-oriented DS MoSi_2_/Mo_5_Si_3_ eutectic composites depends on the growth rate during directional solidification (Section 3.2.1) and is considered to correspond to the stress level at which plastic deformation is initiated in the MoSi_2_ matrix (Section 3.3.1). The yield stress of [1$\bar{1}$0]_MoSi2_-oriented DS MoSi_2_/Mo_5_Si_3_ eutectic composites is thus considered to vary primarily with the average thickness of the MoSi_2_ matrix. Values of yield stress of [1$\bar{1}$0]_MoSi2_-oriented binary and some ternary DS eutectic composites are plotted in Figure 11 as a function of the average thickness of MoSi_2_.

For the binary alloys grown at 100 mm h^–1^ with a (non-homogeneous) cellular structure, the average thickness values of the (fine) central regions, (coarse) cell-boundary regions and overall regions are indicated in the figure. The yield stresses for those with a homogeneous script lamellar structure increase with the decrease in the average thickness of MoSi_2_ both at 1000 and 1400 °C with the decrease being more pronounced at 1400 °C. Microstructure refinement of DS MoSi_2_/Mo_5_Si_3_ eutectic composites is thus quite effective in obtaining better high-temperature strength.

The trend for the MoSi_2_ average thickness dependence of yield stress is very similar for binary and all ternary alloys at both 1000 and 1400 °C, except for that alloyed with 5 at.% Ta tested at 1400 °C. The yield stress at 1400 °C for the 5 at.% Ta-doped alloy is significantly higher than those of binary and other ternary alloys, exceeding that of [001]-oriented single crystals of binary Mo_5_Si_3_. Ta additions are thus found to be very effective in obtaining better high-temperature strength of the DS MoSi_2_/Mo_5_Si_3_ eutectic composites. The substantial increase in yield strength observed for the Ta-doped alloy is believed to be due to the solid-solution hardening effects of large and heavy Ta atoms on plastic deformation of the MoSi_2_ matrix especially at temperatures where diffusion-controlled deformation processes play an important role. This is currently under investigation in our group.

**Figure 12.**
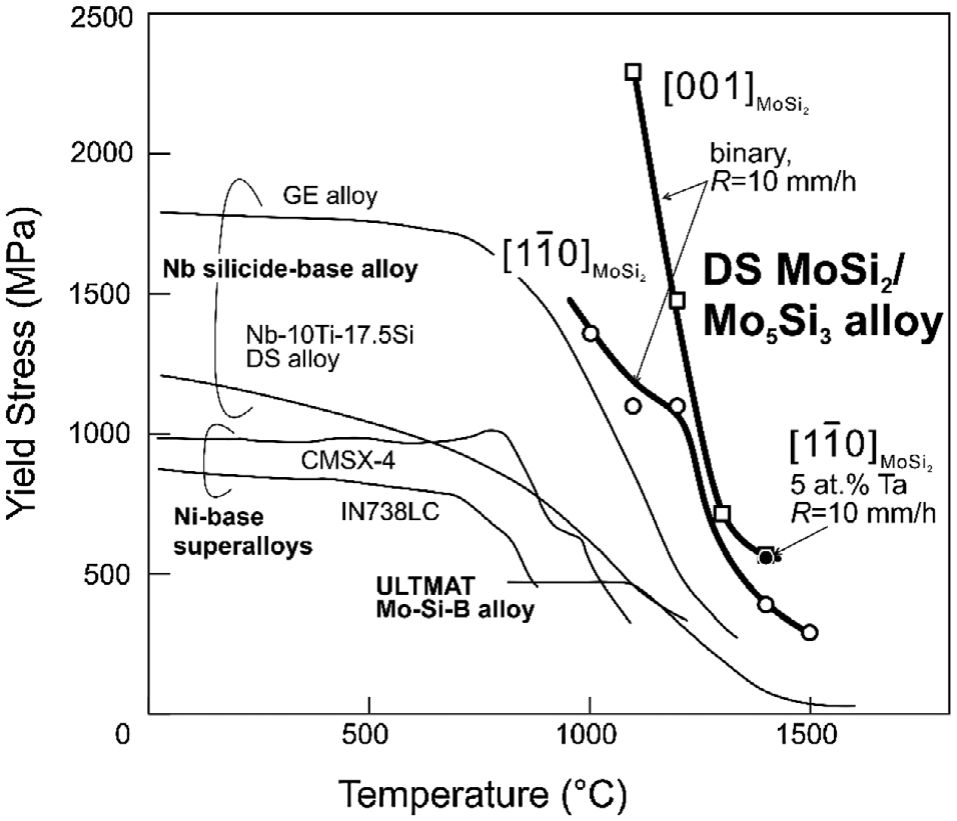
Temperature dependence of yield stress of DS MoSi_2_/Mo_5_Si_3_ eutectic composites and some high-temperature materials.[35–39]

Figure 12 shows the temperature dependence of yield stress for [1$\bar{1}$0]_MoSi2_ and [001]_MoSi2_-oriented binary DS MoSi_2_/Mo_5_Si_3_ eutectic composites grown at 10 mm h^–1^ together with that for [1$\bar{1}$0]_MoSi2_-oriented Ta-doped DS eutectic composites grown at 10 mm h^–1^. The temperature dependence of yield stress obtained for some typical high-temperature materials [35–39] are also shown in the figure for comparison. [001]_MoSi2_-oriented DS eutectic composites exhibit yield stress values higher than [1$\bar{1}$0]_MoSi2_-oriented DS eutectic composites at all temperatures investigated (above 1100 °C). Surprisingly, the yield stress value obtained at 1400 °C for the [1$\bar{1}$0]_MoSi2_-oriented Ta-doped DS eutectic composite is comparable to that obtained at 1400 °C for [001]_MoSi2_-oriented DS eutectic composites, exceeding 500 MPa even at such a high temperature of 1400 °C. The yield stress values of these DS MoSi_2_/Mo_5_Si_3_ alloys are much higher than those not only of modern Ni-base superalloys such as CMSX-4 but also of recently developed ultrahigh-temperature structural materials such as ULTMAT Mo-Si-B alloys and Nb silicide-based DS alloys.[35–39] The yield stress values of these DS MoSi_2_/Mo_5_Si_3_ eutectic ingots at 1400 °C are comparable to or higher than those of CMSX-4 at ~1050 °C, which indicates the significant advantage of these DS MoSi_2_/Mo_5_Si_3_ eutectic alloys for high-temperature structural applications over advanced Ni-base superalloys and recently developed ultrahigh-temperature structural materials. Our preliminary results indicate that these DS MoSi_2_/Mo_5_Si_3_ eutectic alloys exhibit excellent creep properties at temperatures exceeding 1300 °C. This will be published soon elsewhere.

## 5. Conclusions

High-temperature mechanical properties as well as deformation mechanisms of DS ingots of binary and some ternary MoSi_2_/Mo_5_Si_3_ eutectic composites grown at various growth rates have been investigated in compression at temperatures from 900 to 1500 °C. The results obtained are summarized as follows.

1. Plastic flow of DS ingots of MoSi_2_/Mo_5_Si_3_ eutectic composites is observed only above 1000 °C when the loading axis is parallel to [1$\bar{1}$0]_MoSi2_, while when the loading axis is parallel to [001]_MoSi2_, it is observed above 1100 °C, which is ~100 °C higher than that for the [1$\bar{1}$0]_MoSi2_ orientation. The difference in the onset temperature for plastic flow for the two orientations has been interpreted in terms of strain compatibility at the interphase boundary by taking into account of the operative deformation modes in the relevant phases.
2. [1$\bar{1}$0]_MoSi2_-oriented binary DS ingots exhibit values of yield stress about 3–10 times higher than those of [1$\bar{1}$0]-oriented MoSi_2_ single crystals in the temperature range from 1000 to 1500 °C. On the other hand, [001]_MoSi2_-oriented binary DS ingots exhibit values of yield stress higher than those of MoSi_2_ single crystals with the corresponding orientation at 1100 °C, but the opposite is true at higher temperatures above 1300 °C.
3. The yield stress values of binary and some ternary DS MoSi_2_/Mo_5_Si_3_ eutectic composites increase with the decrease in the average thickness of MoSi_2_ phase with the trend being much more enhanced at higher temperatures, indicating that microstructure refinement is effective in obtaining better high-temperature strength for these DS eutectic composites.
4. Among the four ternary alloying elements tested (V, Nb, Ta and W), Ta is found to be the most effective in improving yield strength of DS ingots of MoSi_2_/Mo_5_Si_3_ eutectic composites at higher temperatures around 1400 °C.
5. The yield stress values of these DS MoSi_2_/Mo_5_Si_3_ alloys at high temperatures are much higher than those not only of modern Ni-base superalloys such as CMSX-4 but also of recently developed ultrahigh-temperature structural materials such as ULTMAT Mo-Si-B alloys and Nb silicide-based DS alloys.

## Disclosure statement

No potential conflict of interest was reported by the authors.

## Funding

This work was supported by the Advanced Low Carbon Technology Research and Development Program (ALCA) from the Japan Science and Technology Agency (JST), the Elements Strategy Initiative for Structural Materials (ESISM) from Ministry of Education, Culture, Sports, Science and Technology (MEXT) Japan, Grants-in-Aid for Scientific Research from the MEXT of Japan [grant number 15H02300, 15K14162].

## Footnotes

^1^ The chemical compositions for Ta- and W-alloyed DS ingots analyzed by SEM-EDS are revised from those published in our previous paper.[17] The data in Table 1 are estimated using Ta L and W L lines instead of Ta M and W M lines that seriously overlapped with Si K line resulting in relatively large measurement errors in our previous results.

## References

[CIT0001] Hada S, Tsukagoshi K, Masada J (2012). Test results of the world’s first 1,600 °C J-series gas turbine. Mitsubishi Heavy Ind. Tech. Rev.

[CIT0002] Maloy SA, Heuer AH, Lewandowski J (1991). Carbon additions to molybdenum disilicide: improved high-temperature mechanical properties. J. Am. Ceram. Soc.

[CIT0003] Vasudévan AK, Petrovic JJ (1992). A comparative overview of molybdenum disilicide composites. Mater. Sci. Eng. A.

[CIT0004] Maloy SA, Lewandowski J, Heuer AH (1992). Effects of carbon additions on the high temperature mechanical properties of molybdenum disilicide. Mater. Sci. Eng. A.

[CIT0005] Gibala R, Ghosh AK, Van Aken DC (1992). Mechanical behavior and interface design of MoSi_2_-based alloys and composites. Mater. Sci. Eng. A.

[CIT0006] Mason DP, Van Aken DC (1993). The effect of microstructural scale on hardness of MoSi_2_-Mo_5_Si_3_ eutectics. Scripta metall. mater.

[CIT0007] Ito K, Inui H, Shirai Y (1995). Plastic deformation of MoSi_2_ single crystals. Philos. Mag. A.

[CIT0008] Ito K, Yano T, Nakamoto T (1997). Microstructure and mechanical properties of MoSi_2_ single crystals and directionally solidified MoSi_2_-Based alloys. Prog. Mater. Sci.

[CIT0009] Petrovic JJ, Vasudevan AK (1999). Key developments in high temperature structural silicides. Mater. Sci. Eng. A.

[CIT0010] Inui H, Ishikawa K, Yamaguchi M (2000). Effects of alloying elements on plastic deformation of single crystals of MoSi_2_. Intermetallics.

[CIT0011] Inui H, Ishikawa K, Yamaguchi M (2000). Creep deformation of single crystals of binary and some ternary MoSi_2_ with the C11_b_ structure. Intermetallics.

[CIT0012] Mitra R (2006). Mechanical behaviour and oxidation resistance of structural silicides. Int. Mater. Rev.

[CIT0013] Berztiss DA, Cerchiara RR, Gulbransen EA (1992). Oxidation of MoSi_2_ and comparison with other silicide materials. Mater. Sci. Eng. A.

[CIT0014] Mason DP, Van Aken DC (1995). On the creep of directionally solidified MoSi_2_-Mo_5_Si_3_ eutectics. Acta metall. mater.

[CIT0015] Mason DP, Van Aken DC, Mansfield JF (1995). On the microstructure and crystallography of directionally solidified MoSi_2_-Mo_5_Si_3_ eutectics. Acta Metall. Mater.

[CIT0016] Fujiwara K, Sasai Y, Kishida K (2013). Effects of ternary additions on the microstructure of directionally-solidified MoSi_2_/Mo_5_Si_3_ eutectic composites.

[CIT0017] Fujiwara K, Matsunoshita H, Sasai Y (2014). Effects of ternary additions on the microstructure and thermal stability of directionally-solidified MoSi_2_/Mo_5_Si_3_ eutectic composites. Intermetallics.

[CIT0018] Matsunoshita H, Fujiwara K, Sasai Y (2015). Microstructures and mechanical properties of MoSi_2_/Mo_5_Si_3_/Mo_5_Si_3_C ternary eutectic composites.

[CIT0019] Matsunoshita H, Fujiwara K, Sasai Y (2016). Orientation relationships, interface structures, and mechanical properties of directionally solidified MoSi_2_/Mo_5_Si_3_/Mo_5_Si_3_C composites. Intermetallics.

[CIT0020] Sasai Y, Inoue A, Fujiwara K (2013). Plastic deformation of directionally-solidified MoSi_2_/Mo_5_Si_3_ eutectic composites.

[CIT0021] Umakoshi Y, Sakagami T, Hirano T (1990). High temperature deformation of MoSi_2_ single crystals with the C11_b_ structure. Acta metall. mater.

[CIT0022] Kimura K, Nakamura M, Hirano T (1990). High temperature deformation behaviour of MoSi_2_ and WSi_2_ single crystals. J. Mater. Sci.

[CIT0023] Maloy SA, Mitchell TE, Heuer AH (1995). High temperature plastic anisotropy in MoSi_2_ single crystals. Acta metall. mater.

[CIT0024] Ito K, Matsuda K, Shirai Y (1999). Brittle-ductile behavior of single crystals of MoSi_2_. Mater. Sci. Eng. A.

[CIT0025] Yoshimi K, Yoo MH, Wereszczak AA (2001). Yielding and flow behavior of Mo_5_Si_3_ single crystals. Scripta Mater.

[CIT0026] Yoshimi K, Yoo MH, Wereszczak AA (2002). Deformation behavior of Mo_5_Si_3_ single crystal at high temperatures. Mater. Sci. Eng. A.

[CIT0027] Jackson KA, Hunt JD (1966). Lamellar and rod eutectic growth. Trans. Metall. Soc. AIME.

[CIT0028] Matsunoshita H, Sasai Y, Inoue A

[CIT0029] Kad BK, Vecchio KS, Asaro RJ (1995). On the nature of faults in MoSi_2_. Philos. Mag. A.

[CIT0030] Ito K, Yano T, Nakamoto T (1996). Plastic deformation of MoSi_2_ and WSi_2_ single crystals and directionally solidified MoSi_2_-based alloys. Intermetallics.

[CIT0031] Guder S, Bartsch M, Messerschmidt U (2002). Transmission electron microscopy analysis of planar faults on (001) planes in MoSi_2_ single crystals. Philos. Mag. A.

[CIT0032] Ito K, Yano T, Nakamoto T (1999). Plastic deformation of single crystals of WSi_2_ with the C11_b_ structure. Acta Mater.

[CIT0033] Hook RE, Hirth JP (1967). The deformation behavior of isoaxial bicrystals of Fe-3%Si. Acta Metall.

[CIT0034] Kishida K, Inui H, Yamaguchi M (1998). Deformation of lamellar structure in TiAl-Ti_3_Al two-phase alloys. Philos. Mag. A.

[CIT0035] Sengupta A, Putatunda SK, Bartosiewicz L (1994). Tensile behavior of a new single-crystal nickel-based superalloy (CMSX-4) at room and elevated temperatures. J. Mater. Eng. Performance.

[CIT0036] Balikci E, Mirshams RA, Raman A (2000). Tensile strengthening in the nickel-base superalloy IN738LC. J. Mater. Eng. Performance.

[CIT0037] Bewlay BP, Jackson MR, Zhao JC (2003). Ultrahigh-temperature Nb-silicide-based composites. MRS Bull.

[CIT0038] Drawin S, Heilmaier M, Jehannno MP (2006). The EU-funded “ULTMAT” project: ultra high temperature materials for turbines.

[CIT0039] Sekido N, Kimura Y, Miura S (2006). Fracture toughness and high temperature strength of unidirectionally solidified Nb-Si binary and Nb-Ti-Si ternary alloys. J. Alloys Compd.

